# Elevated blood acetoacetate levels reduce major adverse cardiac and cerebrovascular events risk in acute myocardial infarction

**DOI:** 10.1515/med-2023-0793

**Published:** 2023-08-31

**Authors:** Jun Sato, Kosaku Kinoshita, Atsushi Sakurai

**Affiliations:** Division of Emergency and Critical Care Medicine, Department of Acute Medicine, Nihon University School of Medicine, 30-1 Oyaguchi Kamimachi, Itabashi-ku, Tokyo 173-8610, Japan

**Keywords:** acetoacetates, β-hydroxybutyrate dehydrogenase, heart failure, ketone bodies, myocardial infarction

## Abstract

Although elevated blood ketone body levels reduce major adverse cardiac and cerebrovascular events (MACCEs) risk in chronic heart failure, their relationship with acute myocardial infarction remains unknown. We investigated this relationship in patients with acute myocardial infarction. This single-institution retrospective observational study analyzed data from 114 patients with acute myocardial infarction at Nihon University Hospital from May 1, 2018, to November 1, 2022. The cut-off value of acetoacetate for the incidence of in-hospital MACCE was determined by drawing a receiver operating characteristic curve (ROC) and defining patients with acetoacetate above and below the optimal cut-off point value as ROC and low-acetoacetate (LA) groups, respectively. Propensity score matching was performed between the LA and high-acetoacetate (HA) groups. Sex, peak creatine kinase, lactate, and blood glucose were defined as confounding factors between in-hospital MACCEs and acetoacetate, and 1:1 propensity score matching between the LA and HA groups was used, resulting in 40 patients from both groups enrolled in the analysis. There was a significantly lower incidence of in-hospital MACCEs in the HA group (LA group: 9 [22%] vs HA group: 1 [3%], *P* = 0.014). In conclusion, in acute myocardial infarction, elevated blood acetoacetate levels reduce the risk of MACCE.

## 1 Introduction

The heart uses several energy sources, including fatty acids, glucose, lactate, amino acids, and ketone bodies, to produce ATP. The healthy adult heart obtains 60–80% of its energy from fatty acids and 20–40% from glucose [1]. Additionally, it rapidly switches between these energy sources, depending on hormonal factors [2]. During myocardial ischemia, the oxygen supply to the myocardial mitochondria is reduced, and glucose, which is more efficient for producing ATP than fatty acids, becomes the primary energy source for the heart [3]. Some studies have reported that blood ketone body (acetoacetate and β-hydroxybutyrate) levels increase during acute myocardial infarction [4]. Severe chest pain caused by acute myocardial infarction increases endogenous catecholamine, cortisol, and glucagon levels [5], which activate hormone-sensitive lipase and metabolize triglycerides into free fatty acids and glycerol [6]. Free fatty acids are metabolized into ketone bodies in the liver and transported to peripheral tissues. Finally, ATP is produced in the mitochondria through the tricarboxylic acid cycle [7]. We considered that ketone bodies require less oxygen than fatty acids to produce 1 kcal of energy [8]. Thus, ketone bodies may produce more ATP than fatty acids, even during myocardial ischemia with a low oxygen supply. To date, this phenomenon has not been sufficiently investigated. Additionally, the relationship between blood ketone body levels and major adverse cardiac and cerebrovascular events (MACCEs) in patients with acute myocardial infarction remains unknown. Therefore, this study aimed to investigate the relationship between ketone body levels and MACCE incidence after the onset of acute myocardial ischemia.

## 2 Methods

### 2.1 Study population

**Figure 1:**
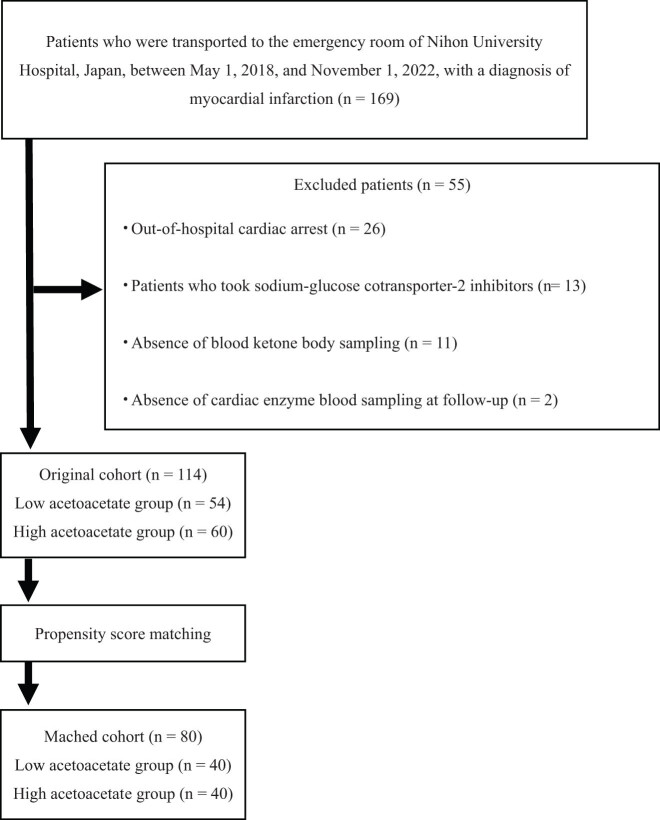
Flow diagram for study participant selection.

This study was designed as a single-institution, retrospective observational investigation of data from 169 patients treated for acute myocardial infarction at Nihon University Hospital from May 1, 2018, to November 1, 2022. A flow diagram of the study participants’ selection process is shown in Figure 1. Fifty-five patients were excluded for the following reasons: 26 out-of-hospital patients experienced cardiopulmonary arrest, 13 received sodium-glucose cotransporter-2 (SGLT2) inhibitors, 11 did not undergo arterial blood ketone body (acetoacetate and β-hydroxybutyrate) measurements at admission, 2 were not followed up for cardiac enzyme levels (creatine kinase [CK], creatine kinase myocardial band [CK-MB], and troponin I [TnI]), and 3 were transferred to another hospital prior to coronary angiography (CAG). Finally, 114 patients were enrolled in this study.

Although early and effective treatment of acute myocardial infarction with percutaneous coronary intervention (PCI) or coronary artery bypass grafting (CABG) can reduce mortality, adverse events, such as stroke or recurrent myocardial infarction, can hamper a patient’s activities of daily living. Therefore, MACCEs during hospitalization were analyzed as an endpoint. In-hospital MACCEs included all-cause mortality, non-fatal myocardial infarction, and non-fatal stroke. The cut-off value of acetoacetate to predict the incidence of in-hospital MACCE was determined by plotting a receiver operating characteristic curve (ROC) and defining patients with acetoacetate above the optimal cut-off point value as the high-acetoacetate group and those below it as the low-acetoacetate group. Propensity score matching was performed between the low-acetoacetate (LA) and high-acetoacetate (HA) groups.

Myocardial infarction was defined based on the Fourth Universal Definition of Myocardial Infarction published in 2018 [9]. The following patient characteristics were examined: age, sex, body mass index, vital signs, comorbidities, medication use, and echocardiographic parameters. Laboratory data included CK, CK-MB, TnI, arterial blood gas, and arterial blood ketone body levels at admission. All patients transported to our hospital’s emergency room had an arterial blood ketone body ratio (AKBR), which was measured to indirectly assess hepatocyte viability, including patients with acute myocardial infarction. The patients underwent CAG on admission. If the coronary culprit lesion was treatable by PCI, they underwent PCI; otherwise, they underwent CABG. The antegrade radiocontrast flow of the infarct-related artery was determined through CAG by the operator who used thrombolysis in myocardial infarction criteria. To monitor cardiac enzyme levels during hospitalization, blood samples were collected first at admission (before PCI) and subsequently 3, 6, 9, 12, and 24 h after PCI.

### 2.2 Statistical analyses

Propensity score matching analysis was performed using EZR, and other analyses were performed using IBM SPSS Statistics (Version 16, IBM Corp., Armonk, NY, USA). Skewed data are presented as median (interquartile range [IQR]) and were evaluated using the Mann–Whitney *U* test. Categorical variables are expressed as numbers (%). Categorical variables were evaluated using Fisher’s exact test or the chi-square test. The Spearman rank test (*ρ*) was used to evaluate the relationship among the variables. Statistical significance was defined as a *P* value of <0.05.

The optimal cut-off value was determined based on the minimum value of the square root of [(1 − sensitivity)^2^ + (1 − specificity)^2^], which represents the minimum distance from the top left corner to a point on the ROC curve.

Propensity score matching was performed between the LA and HA groups. To determine the confounding factors between in-hospital MACCEs and acetoacetate use, factors associated with in-hospital MACCEs were first determined using binomial logistic regression analysis. Among these factors, we defined those related to acetoacetate as confounding factors and performed a 1:1 nearest neighbor matching method.


**Registry and registration no. of the study:** Since this was a retrospective study, it was not registered. However, it has been officially approved by the Clinical Research Review Committee of Nihon University School of Medicine (20220603).
**Approval of the research protocol:** This study was approved by the Clinical Research Review Committee of Nihon University School of Medicine (20220603) and conformed to the provisions of the Declaration of Helsinki.
**Informed consent:** Patient consent was not required because of the retrospective design of the study.

## 3 Results

**Figure 2:**
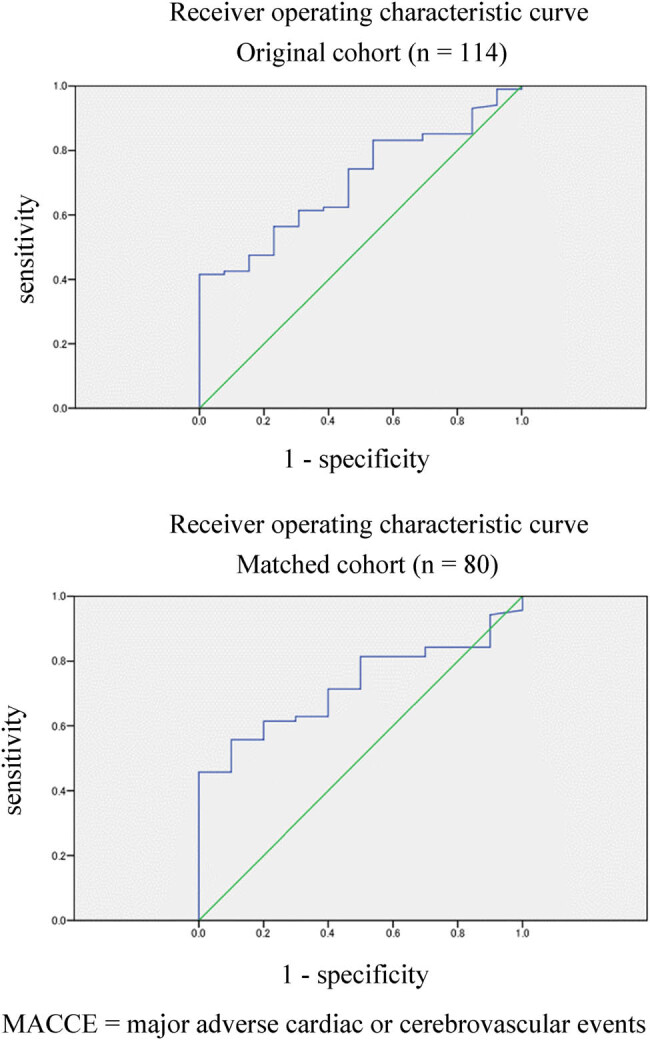
ROCs for acetoacetate to predict the incidence of in-hospital MACCEs. ROC: original cohort (*n* = 114).

In the original cohort, the cut-off value of acetoacetate for in-hospital MACCEs incidence was 130.7 µmol/L, with a sensitivity of 56.4% and a specificity of 76.9% (area under the curve [AUC]: 0.704, standard error [SE] of AUC: 0.062, *P* = 0.017, 95% confidence interval [CI]: 0.584–0.825) (Figure 2). There were 54 cases in the LA group and 60 cases in the HA group above the cut-off value.

We then determined the confounding factors between in-hospital MACCEs and acetoacetate. The relationship between the incidence of in-hospital MACCEs and each factor analyzed using logistic regression analysis in the original cohort is presented in Table 1. The univariate analysis revealed significant differences in the left ventricular ejection fraction (LVEF) on admission, peak CK, peak CK-MB, peak TnI, lactate level, blood glucose level, and acetoacetate level. Of these factors, LVEF was excluded from the confounding factors due to its small correlation coefficient with acetoacetate (*ρ* = −0.013, *P* = 0.891). Peak CK-MB and peak TnI were significantly correlated with peak CK (*ρ* = 0.906, *P* < 0.001 and *ρ* = 0.428, *P* < 0.001, respectively) and were excluded from the confounding factors because of multicollinearity. We added sex to the confounding factors, as it is an important factor. As a result, sex, peak CK, lactate, and blood glucose were defined as confounding factors between in-hospital MACCEs and acetoacetate, and 1:1 propensity score matching between the LA and HA groups was used, resulting in 40 patients from both groups enrolled in the analysis.

**Table 1:** Odds ratios from univariate logistic regression analyses of baseline characteristics associated with in-hospital MACCEs (original cohort, *n* = 114)

Patient characteristics	OR	95% CI	*P*-value
Age	1.025	0.980–1.073	0.276
Male	1.161	0.130–10.410	0.894
BMI	0.977	0.828–1.153	0.786
MAP	0.987	0.962–1.012	0.303
Heart rate	0.986	0.959–1.013	0.307
LVEF on admission	0.923	0.866–0.983	0.013*
Hypertension	0.592	0.185–1.900	0.378
Diabetes mellitus	0.421	0.109–1.625	0.209
Dyslipidemia	0.331	0.101–1.090	0.069
Previous MI	0.785	0.160–3.837	0.765
Current smoker	0.531	0.154–1.838	0.318
T-bil	2.119	0.424–10.584	0.360
AST	1.003	0.995–1.010	0.508
ALT	1.005	0.990–1.020	0.503
Peak CK	1.000	1.000–1.000	0.010*
Peak CK-MB	1.002	1.000–1.004	0.036*
Peak TnI	1.006	1.002–1.010	0.006*
LDL-C	0.996	0.982–1.010	0.555
TG	0.999	0.995–1.003	0.722
eGFR	0.992	0.972–1.013	0.454
Alb	0.554	0.245–1.254	0.157
Lactate	1.280	1.057–1.549	0.011*
Blood glucose	1.006	1.000–1.011	0.045*
AKBR	0.608	0.104–3.563	0.582
AcAc	0.991	0.982–0.999	0.037*
βOHB	0.998	0.995–1.001	0.119

*P < 0.05, BMI = body mass index, GCS = Glasgow coma scale, MAP = mean arterial pressure, LVEF = left ventricular ejection fraction, MI = myocardial infarction, T-bil = total bilirubin, AST = aspartate aminotransferase, ALT = alanine aminotransferase, CK = creatine kinase, CK-MB = creatine kinase myocardial band, TnI = troponin I, LDL-C = low-density lipoprotein cholesterol, TG = triglycerides, eGFR = estimated glomerular filtration rate, Alb = albumin, AKBR = arterial blood ketone body ratio, AcAc = acetoacetate, βOHB = 3-hydroxybutyric acid, OR = odds ratio, 95% CI = 95% confidence interval.

The baseline characteristics, laboratory data, procedural characteristics, and outcomes for the matched and original cohorts are presented in Table 2 and Table S1, respectively. The characteristics of the matched cohort are as follows. There was no significant difference in the median age between the LA and HA groups (LA group: 65 years [IQR: 55–71 years] vs HA group: 65 years [IQR: 53–74 years]; *P* = 0.912). There were no significant differences in the confounding factors used for propensity score matching (sex, peak CK, lactate, and blood glucose) between the two groups. AKBR was significantly lower in the HA group (LA group: median 0.82 [IQR: 0.57–1.13] vs HA group: median 0.53 [IQR: 0.46–0.64]; *P* < 0.001). There was a significantly lower incidence of in-hospital MACCEs in the HA group (LA group: 9 [22%] vs HA group: 1 [3%], *P* = 0.014). In the matched cohort, we plotted ROC curves for acetoacetate to predict the incidence of in-hospital MACCEs (Figure 2). The cut-off value of acetoacetate to predict the incidence of in-hospital MACCE in the original cohort (130.7 µmol/L) had a sensitivity of 55.7% and a specificity of 90.0% in the matched cohort (AUC: 0.724, SE of AUC: 0.063, *P* = 0.023, 95% CI: 0.601–0.846).

**Table 2:** Baseline patient characteristics, laboratory data, procedural characteristics, and outcomes of the matched cohort (*n* = 80)

Patient characteristics	LA group (*n* = 40)	HA group (*n* = 40)	*P*-value
Age, years	65 (55–71)	65 (53–74)	0.912
Male, *n* (%)	34 (85)	33 (83)	1.000
BMI, (kg/m^2^)	24 (22–26)	23 (20–26)	0.210
MAP, (mmHg)	109 (96–124)	109 (87–120)	0.197
Heart rate, beats/min	71 (61–85)	74 (67–87)	0.433
LVEF on admission, (%)	47 (40–55)	45 (40–59)	0.729
**Laboratory data on admission**			
Peak CK, (U/L)	1580 (505–3833)	1818 (729–2886)	0.829
Peak CK-MB, (U/L)	135 (44–373)	159 (67–284)	0.788
Peak TnI, (ng/mL)	45 (16–123)	62 (25–113)	0.679
HDL-C, (mg/dL)	45 (39–50)	50 (43–55)	0.070
LDL-C, (mg/dL)	117 (100–150)	130 (101–149)	0.513
Lactate, (mmoL/L)	1.9 (1.4–2.2)	1.6 (1.2–2.5)	0.620
Blood glucose, (mg/dL)	143 (128–191)	146 (119–173)	0.501
AKBR	0.82 (0.57–1.13)	0.53 (0.46–0.64)	< 0.001*
AcAc	57 (44–85)	239 (148–359)	< 0.001*
βOHB	74 (33–179)	465 (283–664)	< 0.001*
**Myocardial type**			
STEMI, *n* (%)	32 (80)	35 (88)	0.0546
NSTEMI, *n* (%)	8 (20)	5 (13)	0.0546
**Treatment**			
PCI, *n* (%)	38 (95)	38 (95)	1.000
Door-to-balloon time, min	86 (61–118)	78 (60–98)	0.962
CABG, *n* (%)	1 (2)	3 (8)	0.615
**Outcome**			
In-hospital MACCE, *n* (%)	9 (22)	1 (3)	0.014*
In-hospital all-cause mortality, *n* (%)	4 (10)	1 (2.5)	0.359
In-hospital non-fatal MI, *n* (%)	4 (10)	1 (2.5)	0.359
In-hospital non-fatal stroke, *n* (%)	2 (5)	0 (0)	0.494
In-hospital serious ventricular arrhythmia, *n* (%)	4 (10)	2 (5)	0.675
Length of hospital stay, days	13 (10–19)	12 (10–16)	0.488

LA group = low-acetoacetate group (acetoacetate levels ≤ 130.7 mmol/L); HA group = high-acetoacetate group (acetoacetate levels > 130.7 mmol/L). *P < 0.05. Data are presented as number (%) or median (IQR). BMI = body mass index, MAP = mean arterial pressure, LVEF = left ventricular ejection fraction, CK = creatine kinase, CK-MB = creatine kinase myocardial band, TnI = troponin I, HDL-C = high-density lipoprotein cholesterol, LDL-C = low-density lipoprotein cholesterol, AKBR = arterial blood ketone body ratio, AcAc = acetoacetate, βOHB = 3-hydroxybutyric acid, STEMI = ST-elevation myocardial infarction, NSTEMI = non-ST-elevation myocardial infarction, PCI = percutaneous coronary intervention, CABG = coronary artery bypass grafting, MACCE = major adverse cardiac or cerebrovascular events, MI = myocardial infarction, IQR = interquartile range.

## 4 Discussion

Blood ketone bodies are elevated in acute myocardial infarction and chronic heart failure [4,10]. However, the role of blood ketone bodies in acute myocardial infarction remains unknown. To the best of our knowledge, this is the first study to analyze the effect of blood ketone bodies on in-hospital MACCE incidence in patients with acute myocardial infarction. In this study, the significantly lower incidence of in-hospital MACCEs in the high-acetoacetate group suggests that acetoacetate influences the in-hospital MACCEs’ incidence. This may be because acetoacetate is an important energy source during myocardial ischemia.

**Figure 3:**
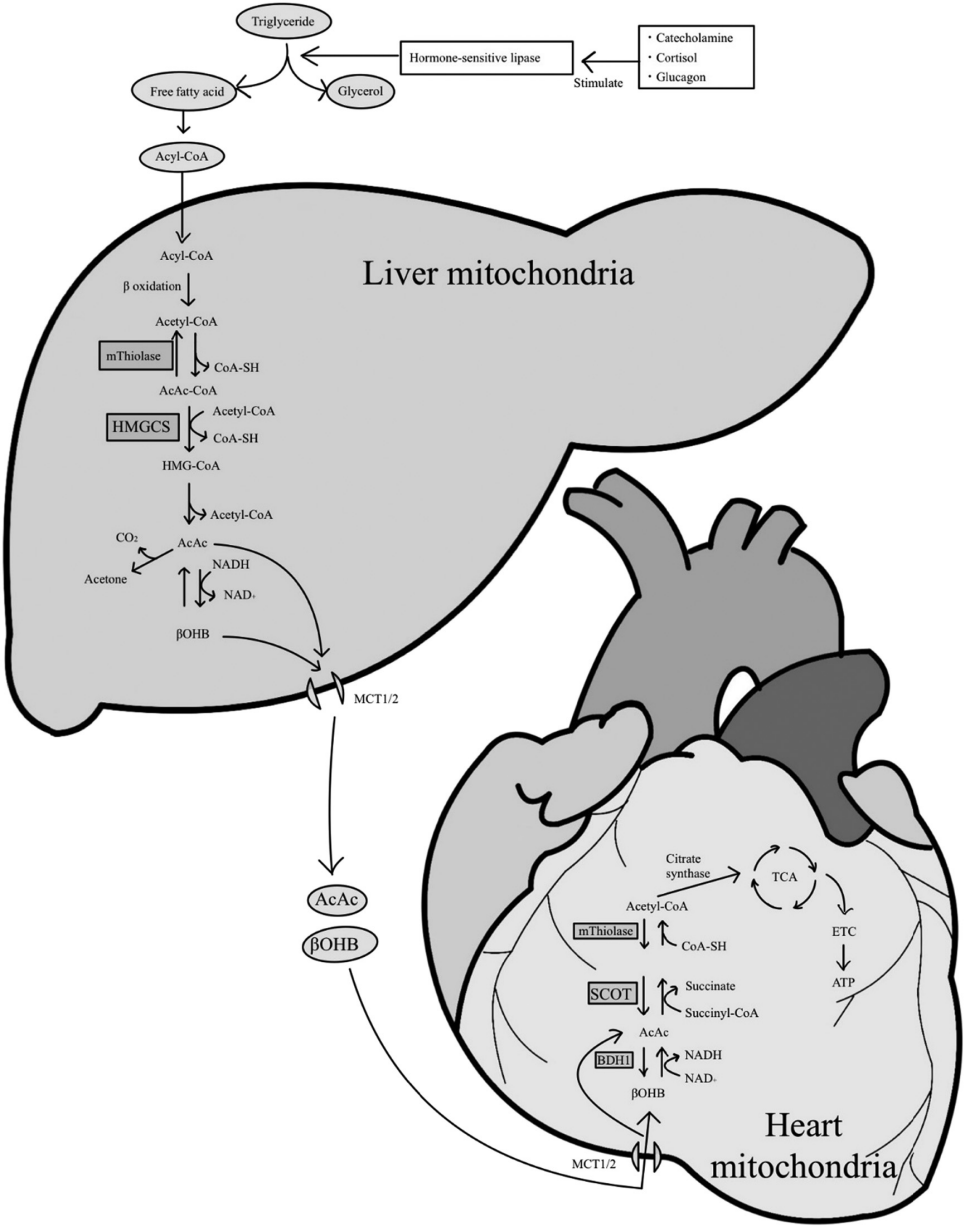
Hypothesized ketone body metabolic pathway. CoA = coenzyme A, AcAc = acetoacetate, HMGCS = hydroxymethylglutaryl-coenzyme A synthase, HMG = hydroxymethylglutaryl, NADH = nicotinamide adenine dinucleotide hydrate, NAD+ = nicotinamide adenine dinucleotide, βOHB = 3-hydroxybutyric acid, MCT = mitochondria through monocarboxylate transporters, βDH1 = β-hydroxybutyrate dehydrogenase, SCOT = succinyl-CoA:3-ketoacid CoA transferase, TCA = tricarboxylic acid cycle, ETC = electron transport chain, ATP = adenosine triphosphate.

Acetoacetate and β-hydroxybutyrate are the two main types of ketone bodies. The third type is acetone, which is spontaneously metabolized from acetoacetate and exhaled; therefore, it has not been discussed in this study. Ketone bodies are produced in the liver and used in peripheral tissues under conditions of limited carbohydrate availability, such as fasting and starvation, exercise, and insulin failure [11]. The metabolic pathways of ketone bodies are shown in Figure 3. Ketone bodies transported to peripheral tissues enter the mitochondria through monocarboxylate transporters (MCTs) present on the inner membrane of the peripheral tissue mitochondria. MCTs transport ketone bodies into the mitochondria down their concentration gradient. When the blood ketone concentration is sufficiently high, the ketone body concentration in the peripheral tissue mitochondria increases with the number of MCTs. Subsequently, ketone bodies are converted to acetyl-CoA by β-hydroxybutyrate dehydrogenase (βDH1) and succinyl-CoA:3-ketoacid CoA transferase (SCOT). ATP is produced via the tricarboxylic acid cycle. When ketone bodies are used to produce ATP in myocardial mitochondria, oxygen consumption is lower than that when glucose and fatty acids are used, and the amount of energy produced is similar to that produced using glucose and fatty acids [8]. Therefore, ketone bodies are highly efficient in terms of oxygen consumption during myocardial ischemia, in which the oxygen supply is inadequate and can be efficiently used to produce ATP.

Several studies have shown that blood ketone levels benefit myocardial energy production. SGLT2 inhibitors reduce the risk of MACCEs in patients with chronic heart failure with or without diabetes [12,13]. The mechanism underlying this effect is increased blood ketone levels induced by SGLT2 inhibitors and the efficient production of ATP using these ketones in myocardial mitochondria [14]. Previous studies have reported that the mRNA levels of MCT1, βDH1, and SCOT increased in acute myocardial infarction rat models fed a ketone diet [15] and that those of βDH1 and SCOT also increased in patients with chronic heart failure [16]. This implies that there may be increased ketone body availability in the myocardial mitochondria under these conditions; however, no study has examined this aspect in patients with acute myocardial infarction. Previous studies have reported that rats with elevated blood ketone body levels after short-term fasting and those that received β-hydroxybutyrate after long-term fasting exhibited reduced infarct size after ischemia–reperfusion treatment [17,18].

This study had a significant relationship between acetoacetate levels and in-hospital MACCEs, which was not observed for β-hydroxybutyrate before propensity score matching. This may be because of the balance between acetoacetate and β-hydroxybutyrate production in the liver, which is influenced by endogenous catecholamine stimulation. AKBR, which reflects the redox potential (nicotinamide adenine dinucleotide [NAD]+/NADH) of hepatic mitochondria, is decreased by shock liver and catecholamine stimulation [19,20]. This is due to increased NADH production in the liver mitochondria as well as increased acetoacetate metabolism to β-hydroxybutyrate. In this study, median AKBR before propensity score matching decreased to 0.59 [IQR: 0.49–0.79], suggesting that stimulation of endogenous catecholamine due to myocardial infarction increased acetoacetate metabolism to β-hydroxybutyrate. Therefore, acetoacetate levels were reduced to a greater extent in patients with high catecholamine stimulation, such as those with in-hospital MACCEs.

A key limitation of this study is that arterial blood ketone body levels were measured at admission without considering meal timing; arterial blood ketone body levels might have been elevated if the patient had fasted.

## 5 Conclusions

In patients with acute myocardial infarction, elevated blood acetoacetate levels are associated with a reduced risk of MACCEs. These results indicate that acetoacetate may serve as a potential protective factor in mitigating severe cardiovascular complications following myocardial infarction, possibly because acetoacetate is an important energy source during myocardial ischemia. Further research and investigations are warranted to elucidate the underlying mechanisms and explore the clinical implications of these findings.

## List of abbreviations

CABG: coronary artery bypass grafting
CAG: coronary angiography
CK: creatine kinase
CK-MB: creatine kinase myocardial band
LVEF: left ventricular ejection fraction
MACCEs: major adverse cardiac and cerebrovascular events
MCTs: monocarboxylate transporters
PCI: percutaneous coronary intervention
SCOT: succinyl-CoA:3-ketoacid CoA transferase
SGLT2: sodium-glucose cotransporter-2
TnI: troponin I
βDH1: β-hydroxybutyrate dehydrogenase

## Supplementary Material

Supplementary Table
