# Supplementary material for: Elevated blood acetoacetate levels reduce major adverse cardiac and cerebrovascular events risk in acute myocardial infarction
Source: Open Med (Wars). 2023 Aug 31;18(1):20230793. doi: 10.1515/med-2023-0793 (PMC10487399; doi:10.1515/med-2023-0793)
Supplement: Supplementary Table [file med-2023-0793-sm.pdf]

Supplementary material

Table S1: Baseline patient characteristics, laboratory data, and procedural characteristics of the original cohort (*n* = 114)

Patient characteristics	LA group (*n* = 54)	HA group (*n* = 60)	*P*-value
Age, years	65 (54–72)	65 (55–75)	0.735
Male, *n* (%)	50 (88)	48 (84)	1.000
BMI, kg/m^2	24 (22–26)	22 (20–26)	0.309
MAP, mmHg	104 (89–118)	111 (96–123)	0.099
Heart rate, beats per min	71 (61–85)	81 (69–97)	0.026*
LVEF on admission, %	48 (40–55)	40 (45–55)	0.940
**Laboratory data on admission**			
Peak CK, U/L	1879 (561–4877)	1902 (993–3431)	0.993
Peak CK-MB, U/L	177 (49–394)	177 (85–335)	0.658
Peak TnI, ng/mL	50 (16–146)	76 (28–145)	0.278
HDL-C, mg/dL	46 (40–54)	49 (42–56)	0.254
LDL-C, mg/dL	122 (95–153)	111 (130–149)	0.302
Lactate, mmoL/L	2.1 (1.6–3.9)	1.7 (1.2–2.35)	0.035*
Blood glucose, mg/dL	146 (124–193)	160 (127–215)	0.337
AKBR	0.71 (0.52–0.98)	0.52 (0.47–0.61)	<0.001*
AcAc			<0.001*
βOHB			<0.001*
**Myocardial type**			
STEMI, *n* (%)	48 (84)	49 (86)	0.590
NSTEMI, *n* (%)	9 (16)	8 (14)	0.590
**Treatment**			
PCI, *n* (%)	55 (96)	54 (95)	1.000
Door-to-balloon time, min	84 (57–111)	82 (61–115)	0.333
CABG, *n* (%)	2 (4)	5 (9)	0.443
**Outcome**			
In-hospital MACCE, *n* (%)	10 (17)	3 (5)	0.036*
In-hospital all-cause mortality, *n* (%)	4 (7)	2 (4)	0.420
In-hospital non-fatal MI, *n* (%)	4 (7)	2 (4)	0.4200
In-hospital non-fatal stroke, *n* (%)	3 (5)	0 (0)	0.103

(Continued)

Table S1: *Continued*

Patient characteristics	LA group (*n* = 54)	HA group (*n* = 60)	*P*-value
In-hospital severe ventricular arrhythmia, *n* (%)	7 (12)	1 (2)	0.147
Length of hospital stay, days	13 (11–20)	12 (10–19)	0.091

*$P < 0.05$.

LA group = low acetoacetate group (acetoacetate levels ≤130.7 mmol/L).

HA group = high acetoacetate group (acetoacetate levels >130.7 mmol/L).

Data are presented as number (%) or median (IQR).

BMI = body mass index, MAP = mean arterial pressure, LVEF = left ventricular ejection fraction, MI = myocardial infarction, CK = creatine kinase, CK-MB = creatine kinase myocardial band, TnI = troponin I, HDL-C = high-density lipoprotein cholesterol, LDL-C = low-density lipoprotein cholesterol, AKBR = arterial blood ketone body ratio, AcAc = acetoacetate, βOHB = 3-hydroxybutyric acid, STEMI = ST-elevation myocardial infarction, NSTEMI = non-ST-elevation myocardial infarction, PCI = percutaneous coronary intervention, CABG = coronary artery bypass grafting, MACCE = major adverse cardiac or cerebrovascular events, MI = myocardial infarction, IQR = interquartile range.

Table S2: Additional baseline patient characteristics, laboratory data, and procedural characteristics

Comorbidities	Original cohort (*n* = 114)		*P*-value	Matched cohort (*n* = 80)		*P*-value
	LA group (*n* = 54)	HA group (*n* = 60)		LA group (*n* = 40)	HA group (*n* = 40)	
Hypertension, *n* (%)	33 (61)	41 (75)	0.439	25 (63)	27 (68)	0.815
Diabetes mellitus, *n* (%)	18 (33)	27 (45)	0.251	15 (38)	13 (32)	0.815
Dyslipidaemia, *n* (%)	32 (59)	39 (65)	0.566	24 (60)	25 (62.5)	1.000
Hyperuricaemia, *n* (%)	11 (20)	13 (22)	0.576	7 (18)	10 (25)	0.255
Previous MI, *n* (%)	7 (13)	14 (23)	0.266	5 (13)	8 (29)	0.546
Chronic heart failure, *n* (%)	3 (6)	4 (7)	1.000	2 (5)	3 (8)	1.000
Chronic kidney disease, *n* (%)	7 (13)	9 (15)	0.794	6 (15)	6 (15)	1.000
Current smoker, *n* (%)	21 (39)	29 (48)	0.348	19 (48)	19 (48)	1.000
**Medications on admission**						
Antiplatelet agents, *n* (%)	5 (9)	13 (22)	0.078	4 (10)	8 (20)	0.348
Calcium-channel blocker, *n* (%)	21 (39)	21 (35)	0.701	15 (38)	12 (30)	0.637
Beta-blocker, *n* (%)	6 (11)	9 (15)	0.59	5 (13)	7 (18)	0.755
RAS-I, *n* (%)	20 (37)	20 (33)	0.699	14 (35)	13 (33)	1.000
Statin, *n* (%)	13 (24)	19 (32)	0.409	11 (28)	12 (30)	1.000
Nitrates, *n* (%)	2 (4)	3 (5)	1.000	2 (5)	3 (8)	1.000
Insulin, *n* (%)	3 (6)	5 (8)	0.720	3 (8)	2 (5)	1.000
DPP-4-I, *n* (%)	7 (13)	14 (23)	0.226	6 (15)	5 (13)	1.000
BG, *n* (%)	4 (7)	4 (7)	1.000	4 (10)	2 (5)	0.675
**Laboratory data on admission**						
WBC, $\times 10^3/\mu L$	9.0 (6.8–11.6)	8.9 (7.4–10.9)	0.650	8.6 (6.7–10.6)	9.0 (7.5–10.9)	0.315
Hb, g/dL	14.5 (12.4–15.6)	14.3 (12.7–15.8)	0.768	14.6 (12.5–15.4)	14.1 (12.4–15.8)	0.851
Ht, %	43.3 (37.7–46.8)	43.0 (38.7–47.2)	0.761	43.2 (37.8–46.2)	42.0 (37.8–55.3)	0.878
Plt, $\times 10^9/L$	238 (194–273)	231 (201–272)	0.779	241 (193–273)	233 (205–274)	0.758
D-dimer, μg/mL	0.80 (0.61–1.86)	0.76 (0.51–1.32)	0.154	0.77 (0.61–1.10)	0.66 (0.50–1.35)	0.233
T-bil, mg/dL	0.49 (0.36–0.69)	0.58 (0.44–0.78)	0.05	0.47 (0.36–0.69)	0.59 (0.46–0.79)	0.036*
AST, U/L	32 (23–56)	34 (26–64)	0.545	30.5 (21.8–42.0)	34.5 (29.8–60.5)	0.145
ALT, U/L	25 (18–47)	25 (16–38)	0.534	24.5 (17.8–45.0)	23.5 (17.5–37.8)	0.904
TG, mg/dL	137 (82–204)	116 (75–178)	0.223	140 (93–204)	107 (74–177)	0.094
eGFR, mL/min/1.73 m^2	59.3 (44.8–71.2)	65.6 (43.4–80.8)	0.217	58.2 (45.1–70.9)	63.7 (43.4–78.9)	0.441
Alb, g/dL	4.1 (3.6–4.4)	4.2 (3.9–4.5)	0.150	4.0 (3.6–4.4)	4.2 (4.0–4.6)	0.035*
HCO_3^-, mmoL/L	21.8 (19.3–23.6)	21.7 (19.6–23.3)	0.897	22.3 (20.5–24.3)	21.7 (19.4–23.6)	0.296
**Final TIMI flow in culprit lesion (post-PCI or post-CABG)**						
TIMI 0, *n* (%)	0 (0)	2 (4)	0.633	0 (0)	2 (5)	0.425
TIMI 1, *n* (%)	0 (0)	0 (0)		0 (0)	0 (0)	
TIMI 2, *n* (%)	4 (7)	4 (7)		3 (8)	2 (5)	
TIMI 3, *n* (%)	50 (93)	51 (90)		37 (93)	34 (90)	
**Treatment**						
IABP, *n* (%)	16 (30)	14 (23)	0.525	9 (23)	9 (23)	1.000
VA-ECMO, *n* (%)	5 (10)	2 (3)	0.253	4 (10)	1 (3)	0.359

(Continued)

Table S2: *Continued*

Comorbidities	Original cohort (*n* = 114)		*P*-value	Matched cohort (*n* = 80)		*P*-value
	LA group (*n* = 54)	HA group (*n* = 60)		LA group (*n* = 40)	HA group (*n* = 40)	
Ventilator, *n* (%)	8 (15)	3 (5)	0.112	4 (10)	2 (5)	0.675
Temporary cardiac pacing, *n* (%)	6 (11)	2 (3)	0.147	2 (5)	3 (8)	1.000

*$P < 0.05$.

LA group = low acetoacetate group (acetoacetate levels ≤130.7 mmol/L).

HA group = high acetoacetate group (acetoacetate levels >130.7 mmol/L).

Data are presented as number (%) or median (IQR).

MI = myocardial infarction, RAS-I = renin-angiotensin system inhibitor, DPP4-I = dipeptidyl peptidase-4 inhibitor, BG = biguanide, WBC = white blood cells, Hb = haemoglobin, Ht = haematocrit, Plt = platelet count, T-bil = total bilirubin, AST = aspartate aminotransferase, ALT = alanine aminotransferase, TG = triglycerides, eGFR = estimated glomerular filtration rate, Alb = albumin, IABP = intra-aortic balloon pump, VA-ECMO = venoarterial extracorporeal membrane oxygenation, TIMI = thrombolysis in myocardial infarction, IQR = interquartile range.
